# Effects on Subclinical Heart Failure in Type 2 Diabetic Subjects on Liraglutide Treatment vs. Glimepiride Both in Combination with Metformin: A Randomized Open Parallel-Group Study

**DOI:** 10.3389/fendo.2017.00325

**Published:** 2017-11-14

**Authors:** Thomas Nyström, Irene Santos-Pardo, Fredric Hedberg, Johan Wardell, Nils Witt, Yang Cao, Leif Bojö, Bo Nilsson, Johan Jendle

**Affiliations:** ^1^Department of Clinical Science and Education, Karolinska Institutet, Södersjukhuset, Stockholm, Sweden; ^2^Unit of Biostatistics, Institute of Environmental Medicine, Karolinska Institutet, Stockholm, Sweden; ^3^Clinical Epidemiology and Biostatistics, School of Medical Sciences, Örebro University, Örebro, Sweden; ^4^Central Hospital, Karlstad, Sweden; ^5^Institution of Medical Science, Örebro University, Örebro, Sweden

**Keywords:** longitudinal functional reserve index, liraglutide, subclinical heart failure, tissue Doppler echocardiography, type 2 diabetes

## Abstract

**Objective:**

We aimed to investigate the effect of liraglutide treatment on heart function in type 2 diabetes (T2D) patients with subclinical heart failure.

**Methods:**

Randomized open parallel-group trial. 62 T2D patients (45 male) with subclinical heart failure were randomized to either once daily liraglutide 1.8 mg, or glimepiride 4 mg, both add on to metformin 1 g twice a day. Mitral annular systolic (s′) and early diastolic (e′) velocities were measured at rest and during bicycle ergometer exercise, using tissue Doppler echocardiography. The primary endpoint was 18-week treatment changes in longitudinal functional reserve index (LFRI_diastolic/systolic_).

**Results:**

Clinical characteristics between groups (liraglutide = 33 vs. glimepiride = 29) were well matched. At baseline left ventricle ejection fraction (53.7 vs. 53.6%) and global longitudinal strain (−15.3 vs. −16.5%) did not differ between groups. There were no significant differences in mitral flow velocities between groups. For the primary endpoint, there was no treatment change [95% confidence interval] for: LFRI_diastolic_ (−0.18 vs. −0.53 [−0.28, 2.59; *p* = 0.19]), or LFRI_systolic_ (−0.10 vs. −0.18 [−1.0, 1.7; *p* = 0.54]); for the secondary endpoints, there was a significant treatment change in respect of body weight (−3.7 vs. −0.2 kg [−5.5, −1.4; *p* = 0.001]), waist circumference (−3.1 vs. −0.8 cm [−4.2, −0.4; *p* = 0.019]), and heart rate (HR) (6.3 vs. −2.3 bpm [−3.0, 14.2; *p* = 0.003]), with no such treatment change in hemoglobin A1c levels (−11.0 vs. −9.2 mmol/mol [−7.0, 2.6; *p* = 0.37]), between groups.

**Conclusion:**

18-week treatment of liraglutide compared with glimepiride did not improve LFRI_diastolic/systolic_, but however increased HR. There was a significant treatment change in body weight reduction in favor for liraglutide treatment.

## Introduction

Liraglutide is a glucagon-like peptide receptor agonist (GLP-1RA) approved for the treatment of type 2 diabetes (T2D). Besides lowering, glucose GLP-1RA have other beneficial effects such as weight reduction and low risk of hypoglycemia. Other than glycemic actions also have gained increasing attention, in which potential beneficial role on cardiovascular (CV) function is of high interest (1).

In the Liraglutide Effect and Action in Diabetes trial, which was launched to assess CV safety in T2D patients with high CV risk, treatment with liraglutide was compared with placebo (2). The time-to-event analysis for the composite endpoint, i.e., the rate of the first occurrence of CV death, nonfatal myocardial infarction, or nonfatal stroke was significantly lower among T2D patients treated with liraglutide, compared with placebo. This effect was mainly driven by a significantly lower rate of CV death. The rates of nonfatal myocardial infarction, nonfatal stroke, and hospitalization of heart failure also were, however, nonsignificantly lower in the liraglutide group than in the placebo group (2).

In T2D, diastolic heart failure is common and precedes overt heart failure (3). The detection of diastolic heart failure may provide an approach both for identifying and to treat high-risk individuals who may benefit from earlier and more active intervention to prevent overt heart failure (3). Several small clinical studies have shown beneficial action on systolic heart function after GLP-1 treatment (4, 5); however, recent studies have not been conclusive (6, 7). Notwithstanding this, studies on the treatment of GLP-1RA in T2D patients with subclinical heart failure are scarce (8, 9).

We aimed to investigate whether 18-week treatment of liraglutide, compared with glimepiride, could improve diastolic and or systolic longitudinal functional reserve index (LFRI), using stress exercise tissue Doppler echocardiography (TDE), in T2D patients with subclinical heart failure.

## Materials and Methods

### Trial Design

This is an open, assessor-blinded, randomized, controlled, parallel-group trial. Patients with T2D and subclinical heart failure was block randomized (randomly permuted blocks), with sealed envelope, to receive liraglutide, or glimepiride during 18-week treatment. Patients were recruited from the Endocrinology and Cardiology units of two hospitals (Central Hospital, Karlstad and Södersjukhuset, Stockholm) in Sweden. The trial protocol was reviewed and approved by the regional ethics committee at both participating centers and the study has been carried out following the International Conference on Harmonization-Good Clinical Practice guidelines. All patients provided written informed consent before enrollment. Clinical Trial Registration Information URL: http://www.clinicaltrials.gov. Unique identifier: NCT01425580.

### Subjects

Type 2 diabetes patients who had a glycated hemoglobin A1c (HbA1c) between 45 and 97 mmol/mol were eligible if they had not been previously treated with GLP-1RA, dipeptidyl peptidase-4 inhibitors, or glimepiride. Patients who met these criteria were invited for echocardiographic screening, in which one of the following criteria had to be fulfilled: left ventricle ejection fraction (LVEF) ≤50%, or evidence of diastolic dysfunction as shown by E/e′ ratio > 15, or with signs of increased left atrial size (>49 ml/m^2^), or decreased systolic velocity (s′) ≥ 20% in at least two of four segments compared to normal population (10).

The major exclusion criteria were: type 1 diabetes, treatment with glitazones during the last 6 months, treatment with sulfonylureas the last 3 months or insulin treatment within the last month, heart failure classified according to the New York Heart Association classification (NYHA) 3–4, past history of atrial fibrillation or flutter, presence of acute myocarditis or significant valvulopathies, uncontrolled hypertension, severe heart conduction disturbances or ventricular tachyarrhythmia within the last 3 months, unstable angina or myocardial infarction the previous 8-week, estimated glomerular filtration rate <30 ml/min, hemoglobin <90 g/l, BMI >40 kg/m^2^, severe gastrointestinal disease, history of acute or chronic pancreatitis, malign neoplasia within the last 5 years, current drug or alcohol abuse, and pregnancy.

### Outcomes

The primary endpoint was treatment change in either LFRI_diastolic_ or LFRI_systolic_. LFRI have previously been used to detect subclinical LV dysfunction in patients with diabetes compared to non-diabetic controls (11). Briefly, LFRI was constructed by collecting e′ and s′ at rest (e′_rest_, s′_rest_), and during bicycle exercise stress test (e′_exercise_, s′_exercise_) using the formula: LFRI_diastolic_ = Δe′ × [1 − (1/e′_rest_)], and LFRI_systolic_ = Δs′ × [1 − (1/s′_rest_)], respectively. Where Δe′ is the difference between e′_exercise_ and e′_rest_ and Δs′ is the difference between s′_exercise_ and s′_rest_ (Note that LFRI has no magnitude).

Secondary endpoints were treatment changes in: LVEF, global longitudinal strain (GLS), E/e′ ratio, body weight, waist circumference, systolic and diastolic blood pressure, heart rate (HR), glycemic control measured with HbA1c, and metabolic markers of: lipids, C-reactive protein (CRP), and N-terminal-pro brain natriuretic protein (NT-proBNP).

### Procedures and Treatment

Patients attended a screening visit (visit 1) to assess their eligibility. Patients, if found eligible patients, were scheduled within the next 4 weeks to up-titrate metformin, up to 1 g twice daily, or the maximal tolerated dosage (run-in period). At visit 2, following data were collected: clinical assessment of heart failure according to NYHA classification, anthropometric assessment, blood testing for metabolic parameters, rest TDE, and bicycle ergometer stress test with TDE assessment. Thereafter, patients were randomized to receive the study drug liraglutide, or the comparator treatment glimepiride. For the liraglutide group, the initial dose of liraglutide was 0.6 mg (s.c.) with an up-titration of 0.6 mg every week achieving a final dose of 1.8 mg per day. In the comparator group, 2 mg glimepiride was initially administrated with an up-titration of 1 mg every week reaching a final dose of 4 mg per day. All patients were supplied with a glucometer device (Abbot Contour^®^). All measurements were performed with drawn capillary blood with test strips calibrated to plasma values. The participants were asked to monitor a 7-point profile glucose curve three days before visit 3 and visit 4, which were telephone-visits, and at the end of treatment (visit 5). At visit 5, the patients were re-tested as for visit 2. All patients were offered a final visit (visit 6) in which the anti-diabetic treatment to be followed the trial was decided. Monitoring drug accountability assessed patients’ treatment compliance.

### Clinical Data and Metabolic Parameters

Height, body weight, waist circumference, and clinical data of: diabetes duration, the presence of diabetes-related complications, previous history of CV disease, treatment of hypertension, or dyslipidemia and smoking were collected at visit 2. After 15 min of rest, in a sitting position, measurements of systolic and diastolic blood pressure and HR (systolic and diastolic blood pressure was recorded three times in both arms, simultaneously with measurements of HR), and finally a venipuncture for blood test, were carried out.

### Echocardiographic Parameters and Bicycle Ergometer Stress Test

Transthoracic TDE was performed with a Vivid E9 system (GE, Vingmed Ultrasound, Horten, Norway) with a 3.5 MHz phased-array transducer as well as a triplane 3.5 MHz phased-array transducer. Patients were lying in the left lateral decubitus position in quiet respiration. Cine-loops including at least three consecutive heartbeats were saved and transmitted to a work station (EchoPAC-PC version 60; GE Medical systems, Horten, Norway) for off-line analysis. All off-line analyses were made by one investigator (JW) to reduce variability. Measurements of cardiac dimensions were made in accordance with recommendations of the American Society of Echocardiography Committee (10). Left ventricular ejection fraction was assessed by biplane Simpson’s rule, and GLS were calculated for the entire U-shaped length of LV myocardium (10). Diastolic mitral inflow velocities were obtained from apical 4-chamber view. TDE imaging loops were recorded from apical 2- and 4-chamber views. Pulsed TDE imaging loops, and color-coded TDE imaging recordings were saved. Sector width was optimized for adequate myocardial visualization and frame-rate of far more than 100/s was obtained. Long axes myocardial velocities from the septal, lateral, anterior, and inferior basal parts of the left ventricle were extracted from color-coded TDE imaging recordings. The sampling volume was placed close to the mitral annulus. A mean value from three heart cycles was calculated from each site. A graded bicycle ergometer (Monark 839E, Varberg, Sweden) submaximal test with a stepwise increase of 20 W every other minute, until Borg scale of 15 was reached, was used (12). This was performed using a Case 5 system (GE Medical Systems, WI, USA). Immediately after the submaximal bicycle ergometer stress test, patients were re-investigated with TDE in a lying position.

### Adverse Events

All adverse effects regardless of relationship to study drug, or protocol procedure were registered, reported and monitored. Glycemic reports and adverse effects were evaluated in every visit and when clinically needed. Regarding hypoglycemia episodes, subjects were asked to record all plasma glucose values ≤3.9 mmol/l when hypoglycemic symptoms had occurred.

### Power Calculation

The primary endpoint was an improvement in LFRI_diastolic_ or LFRI_systolic_ measured with TDE. The study was designed to detect an absolute increase in LFRI of 0.7 (i.e., a 15% relative increase in e′ or s′, cm/s) from an assumed mean LFRI_diastolic_ or LFRI_systolic_ of 4 (11). We needed to investigate 42 patients to demonstrate a mean absolute difference in LFRI of 0.7 with an alpha error of 5% and a beta error of 80%. This calculation was done with a SD for the method assumed to be 0.8 cm/s for e′ or s′, respectively.

### Statistical Analysis

Normality of continuous data was checked using Shapiro–Wilk *W* test and homogeneity of variances was checked using Levene’s test. Normally distributed continuous data were summarized as mean ± SD, continuous data with skewed distribution were summarized as median [first quartile (Q1), third quartile (Q3)], and categorical data are presented as percentage. We used Student’s *t*-test, Mann–Whitney *U* test, and Fisher’s exact test to analyze baseline characteristics and treatment change, between groups, for the primary and secondary endpoints. Also, an intention-to-treat analysis was performed after multiple imputations by using iterative Markov chain Monte Carlo method for missing values in multiple continuous variables (13). Five imputed data set were generated and difference between groups was examined using general linear regression model. The estimates from the five data set were combined according to Rubin’s rules. A two-sided *p* value of less than 0.05 was considered statistically significant. Data has been analyzed using Stata 14.2 (StataCorp LLC, College Station, TX, USA).

## Results

### Clinical Characteristics and Echocardiographic Parameters

**Figure 1:**
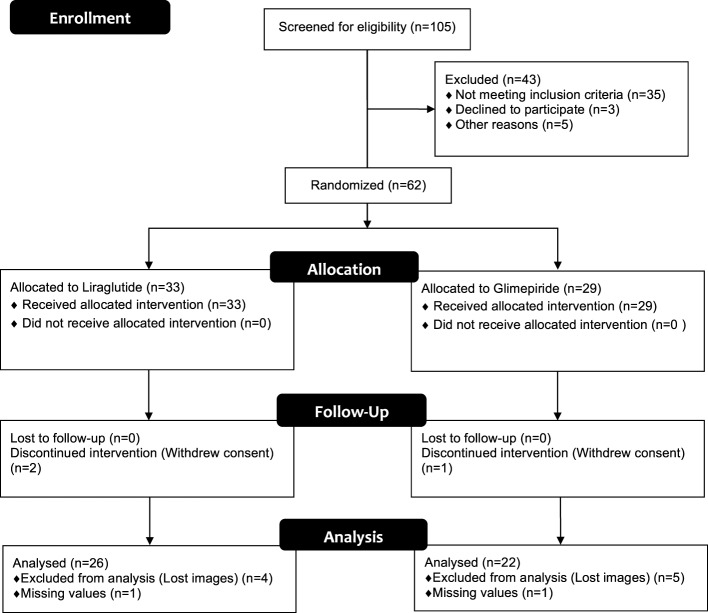
Flow chart for the study groups. 105 patients were screened, whereas 62 patients were eligible for the study, of these 33 vs. 29 were randomized to liraglutide vs. glimepiride, respectively. Due to technical hitches and dropouts, there were 26 in the liraglutide vs. 22 in glimepiride group, who were analyzed per-protocol, i.e., full data set.

A flow chart of the study is given in Figure 1. In total, 105 patients were screened for eligibility, in which 62 met the criteria and were randomized to liraglutide or glimepiride. Due to technical hitches; i.e., TDE images of 9 patients were not correctly spared, and for 2 more patients there were missing TDE data for s′ and e′ at exercise, respectively, and to dropouts; i.e., 3 patients withdrew their informed consent; 2 due to nausea (liraglutide group), and 1 due to lack of time (glimepiride group) 26 in the liraglutide, and 22 in the glimepiride fulfilled the protocol and analyzed per-protocol (Figure 1). After multiple imputations, an intention-to-treat analysis (*n* = 62 patients) was also performed. All clinical baseline characteristics and echocardiographic parameters are given in Table 1. All data, except triglycerides, were normally distributed. Groups were well matched without any significant differences between them (Table 1). According to NYHA classification, there were three patients classified as NYHA II, all other patients were classified as NYHA I. This was further reflected by the mean of EF, which was of 54%, both groups (Table 2).

**Table 1:** Basal clinical characteristics and echocardiographic parameters of the studied T2DM patients.

Basal clinical characteristics	Liraglutide (n = 33)	Glimepiride (n = 29)	p Value
Age, years	61 (7.6)	63 (6.8)	0.240^a^
Male sex	24 (72.7)	21 (72.4)	1.000^b^
Diabetes duration, years	5 (1, 10)	3 (1, 7)	0.368^c^
Smoking	3 (9.1)	4 (13.8)	0.852^b^
BMI, kg/m^2^	30.5 (4.4)	29.0 (3.2)	0.152^a^
Body weight, kg	91.8 (15.9)	89.0 (9.9)	0.411^a^
Waist circumference, cm	109.0 (13.0)	106.3 (9.7)	0.366^a^
Mean systolic BP, mmHg	139 (17)	137 (12)	0.541^a^
Mean diastolic BP, mmHg	83 (9)	83 (8)	0.719^a^
eGFR, ml/min/1.72 m^2^	88.3 (15.0)	87.4 (13.1)	0.654
**Complications**			
Hypertension	29 (87.9)	21 (72.4)	0.224^b^
Hyperlipidemia	25 (75.8)	23 (79.3)	0.980^b^
Coronary artery disease	10 (30.3)	11 (37.9)	0.714^b^
Stroke	1 (3)	2 (6.9)	0.902^b^
Proliferative retinopathy	1 (3)	1 (3.4)	1.000^b^
**Treatment**			
Antiplatelet therapy	11 (33.3)	12 (41.4)	0.696^b^
Anticoagulant treatment	3 (9.1)	1 (3.4)	0.714^b^
ACE inhibitors/ARB blockers	25 (75.8)	20 (69.0)	0.754^b^
Beta-blockers	14 (42.4)	13 (44.8)	1.000^b^
Calcium inhibitors	13 (39.4)	10 (34.5)	0.894^b^
Diuretics	11 (33.3)	6 (20.7)	0.408^b^
Statins	22 (66.7)	24 (82.8)	0.248^b^
**Biochemical parameters**			
HbA1c, mmol/mol	54 (50, 60)	50 (49, 54)	0.036^c^
Triglycerides, mmol/l	2.0 (1.4, 2.6)	1.5 (1.0, 2.2)	0.029^c^
Total cholesterol, mmol/l	4.4 (4.0, 6.0)	4.5 (3.7, 4.8)	0.370^c^
LDL-cholesterol, mmol/l	2.8 (1.2)	2.5 (1.0)	0.440^a^
HDL-cholesterol, mmol/l	1.1 (0.3)	1.2 (0.3)	0.417^a^
CRP, mg/l	2.0 (1.0, 4.0)	1.0 (0.9, 2.4)	0.016^c^
NT-proBNP, mg/l	63 (35, 104)	50 (35, 146)	0.621^c^

**Echocardiographic parameters**	**Liraglutide (*n* = 26)**	**Glimepiride (*n* = 22)**	***p* Value**

**LV and atrial dimensions**			
LVSV, ml	80.2 (17.1)	76.4 (13.4)	0.376^a^
LVED diameter, mm	51.0 (6.5)	48.0 (6.3)	0.096^a^
LVES diameter, mm	37.8 (8.7)	33.8 (8.1)	0.097^a^
Left atrial volume, ml	73.8 (23.9)	54.3 (16.6)	0.013^a^
**Systolic function**			
LVEF at rest, %	53.6 (11.5)	53.7 (9.2)	0.994
GLS at rest, %	−15.3 (4.3)	−16.5 (3.7)	0.319^a^
s′ at rest, cm/s	5.7 (1.1)	5.7 (1.0)	0.918^a^
s′ during exercise, cm/s	8.6 (2.1)	9.4 (1.8)	0.132^a^
LFRI_systolic_^d^	2.4 (1.1)	3.0 (1.3)	0.058^a^
**Diastolic function**			
E-wave at rest, cm/s	66.9 (14.1)	66.8 (14.1)	0.987
e′ at rest, cm/s	5.5 (1.1)	5.5 (1.2)	0.929^a^
E/e′ at rest	12.5 (3.4)	12.3 (2.7)	0.880^a^
e′ during exercise, cm/s	8.0 (1.7)	8.7 (1.7)	0.127^a^
LFRI_diastolic_^d^	2.0 (1.1)	2.6 (1.1)	0.625^a^

*Quantitative data are mean (SD) or median (Q1, Q3), and categorical data are *n* (%)*.

*^a^Student’s t-test was used*.

*^b^Doubled one-sided p value of Fisher’ exact test was used*.

*^c^Mann–Whitney U test was used*.

*^d^Note that LFRI has no magnitude*.

*ARB, angiotensin receptor blockers; BP, blood pressure; CRP, C-reactive protein; E, early diastolic peak velocity; e′, early diastolic mitral annulus velocity; eGFR, estimated glomerular filtration rate; GLS, global longitudinal strain; HbA1c, hemoglobin A1c; HR, heart rate; NT-proBNP, N-terminal pro b-type natriuretic peptide; LFRI, longitudinal function reserve index; LVEF, left ventricular ejection fraction; LVSV, left ventricular stroke volume; LVED, left ventricular end-diastolic; LVES, left ventricular end-systolic; s′, peak systolic annular velocity*.

**Table 2:** Treatment change of secondary outcomes during 18-week treatment.

Clinical characteristics	Liraglutide (*n* = 31)	Glimepiride (*n* = 28)	*p* Value
Body weight, kg	−3.5 (−6,0, −1.1)	0.5 (−1.2, 2.1)	0.001^a^
Waist circumference, cm	−3.1 (2.8)	−0.8 (4.4)	0.019^b^
Mean systolic BP, mmHg	−3.0 (−13.0, 3.0)	−0.5 (−8.5, 8.0)	0.176^a^
Mean diastolic BP, mmHg	−0.3 (8.0)	−0.9 (7.9)	0.761^b^
HR at rest, bpm	6.3 (9.6)	−2.3 (9.4)	0.004^b^
HR during exercise, bpm	−1.0 (−6.0, 8.0)	−4.5 (−12.0, 1.0)	0.150^a^
HbA1c, mmol/mol	−10.0 (−18.0, −4.0)	−5.5 (−12.5, −3.0)	0.112^a^
Triglycerides, mmol/l	−0.2 (0.4)	−0.1 (0.8)	0.492^b^
LDL, mmol/l	−0.1 (−0.5, 0.1)	−0.2 (−0.5, 0.1)	0.994^a^
HDL, mmol/l	0.1 (0.0, 0.2)	0.0 (−0.1, 0.1)	0.386^a^
CRP, mmol/l	0.0 (−1.0, 0.1)	0.0 (0.0, 0.2)	0.351^a^
NT-proBNP, mg/l	−0.0 (−25.0, 11.0)	0.0 (−15.5, 24.5)	0.616^a^

**Echocardiographic parameters**	**Liraglutide (*n* = 26)**	**Glimepiride (*n* = 22)**	***p* Value**

LVEF at rest, %	−2.1 (4.6)	−0.6 (5.9)	0.367
GLS at rest, %	0.0 (2.4)	0.6 (1.8)	0.450^b^
E/e′ at rest	−0.5 (3.1)	−0.4 (2.4)	0.867^b^

*Data are mean (SD) or median (Q1, Q3)*.

*^a^Mann–Whitney U test was used*.

*^b^Student’s t-test was used*.

*BP, blood pressure; CRP, C-reactive protein; E, early diastolic peak velocity; E, early diastolic peak velocity; e′, early diastolic mitral annulus velocity; GLS, global longitudinal strain; HbA1c, hemoglobin A1c; HR, heart rate; LVEF, left ventricular ejection fraction; NT-proBNP, N-terminal pro b-type natriuretic peptide*.

### Bicycle Ergometer Stress Echocardiography

The perceived exertion was set to 15 according to the Borg scale (12). The workload capacity between visit 2 and visit 5 did not differ within groups (liraglutide 115.9 ± 29.4 vs. 115.2 ± 30.4 W, ns; and glimepiride 124.4 ± 29.5 vs. 123.1 ± 29.1 W, ns).

### Primary Outcome

**Figure 2:**
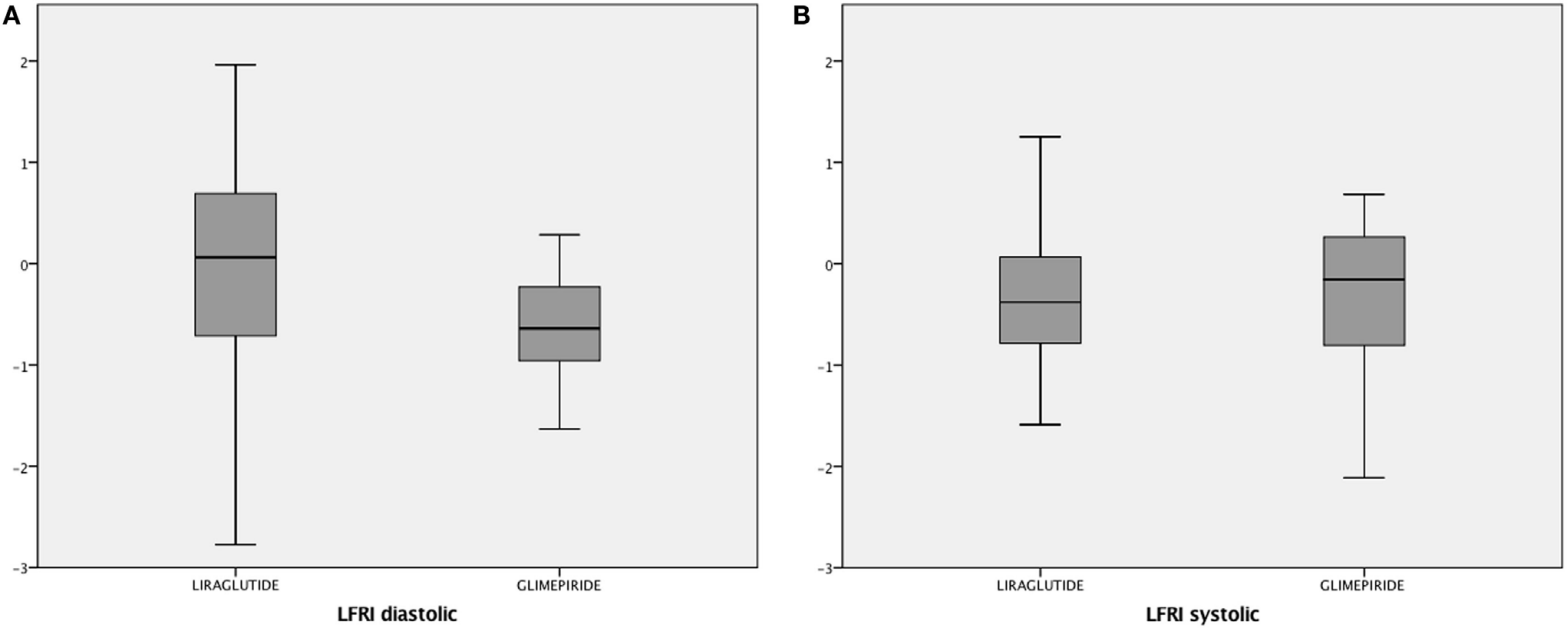
Boxplot representing treatment change, during 18-week treatment of liraglutide vs. glimepiride, for the primary endpoint of diastolic and systolic longitudinal functional reserve index (LFRI_diastolic_ and LFRI_systolic_). Note that LFRI has no magnitude. Shapiro–Wilk *W* test for normality; change in diastolic LFRI liraglutide group, *p* = 0.733; change in diastolic LFRI glimepiride group, *p* = 0.273; change in systolic LFRI liraglutide group, *p* = 0.830; change in systolic LFRI glimepiride group, *p* = 0.182.

There was no treatment change between groups for LFRI_diastolic_ or LFRI_systolic_ (Figure 2). After imputations (intention-to-treat analysis), results did not change from the per-protocol analysis (data not shown).

### Secondary Outcome

All predefined secondary outcomes are given in Table 2. There was a significant treatment change in body weight reduction, and a decrease in waist circumferences in favor of liraglutide treated group. In both groups, there was a robust decrease in HbA1c, however, nonsignificant treatment change for the same (Table 2). There was an increased HR (at rest) in patients treated with liraglutide, compared with glimepiride, without such difference at exercise (Table 2). There was no treatment change for LVEF, GLS, or E/e′, between groups (Table 2). Nor were there any treatment change in CRP levels, or blood lipids, between groups (Table 2). After multiple imputation (intention-to-treat analysis), results did not change from per-protocol analysis (data not shown).

### Adverse Events

We observed the following non-serious gastrointestinal side effects with liraglutide: nausea 8, diarrhea 3, vomiting 1, constipation 1, dizziness 1, urinary tract infection 1, headache 1, and orthostatic reaction 1; and with glimepiride: headache 2, hematuria 1, diarrhea 1, vomiting 1, itching 1, and gingival hyperplasia 1. All adverse events were transient and mild but for two subjects with nausea leading to discontinuation of the study. One severe adverse event (arm fracture leading to surgery) occurred in the liraglutide group. Severe hypoglycemia did not occur in any of the treatment groups. Hypoglycemia occurred in 6 subjects (*n* = 33, 18%) of the patients treated with liraglutide, and in 17 subjects (*n* = 29, 59%) of the patients treated with glimepiride. All the events were mild and documented by self-measured blood glucose.

## Discussion

In the present study, we found that 18-week treatment of liraglutide, compared with glimepiride, did not improve LFRI_diastolic_ or LFRI_systolic_ in T2D patients with subclinical heart failure. There was a robust decrease in HbA1c, in both treated groups, with no treatment change between groups. Liraglutide significantly reduced body weight and waist circumferences, and significantly increased HR, compared with glimepiride.

One novelty in our study was that it was conducted on T2D patients with diastolic dysfunction. The spectrum of diabetic heart disease involves a progression from the normal heart to subclinical diastolic dysfunction followed by clinically overt symptomatic heart failure (3). In our study, patients had no overt symptoms of heart failure, and their mean LVEF was above 50%; although patients had signs of diastolic dysfunction and therefore the development of heart failure with preserved EF (HF-PEF) (13). While a recent published placebo-controlled crossover study failed to show any improvement of systolic function in newly diagnosed T2D patients treated with liraglutide (14), there is some evidence that this drug may have a role in cardiac remodeling diastolic heart function (15, 16). It also was recently demonstrated, in a prospective observational study in patients with T2D, that 6 months liraglutide treatment was associated with a significant improvement in diastolic function concomitant with body weight reduction, although with no correlation between the improvement of the diastolic function and body weight loss (17). The lack of an adequate comparable parallel group in the above study was a major weakness and therefore not easy to interpret (17).

Current study was an open randomized parallel-group study where glimepiride was used as the comparator. This was chosen due to its equal glycemic reduction to liraglutide (18), and that glimepiride may be neutral for heart function (19). In the present study, groups were well matched showing no difference at baseline for the studied parameters; however, there was no treatment change between groups for the primary outcome, except for body weight and waist circumference, which mostly was expected (18). There was an increase in HR in the liraglutide treated group; which also has been observed for the treatment of T2D with GLP-1RA (20), although with some mixed results between types of GLP-1RA (21–23). Our study results contrast with a recent study where 33 T2D patients were treated with liraglutide for 16 weeks compared with placebo add on to supervised exercise. In that study, there was no change in HR between groups; however diastolic function upon supervised exercise was improved in the placebo group and blunted in the liraglutide treated T2D patients (8). In our study, we used bicycle ergometer exercise to sharp the sensitivity of our method, and not for the treatment of diastolic function, therefore studies are not easy to compare. Moreover, in our study, it was an increase in HR (at rest) in the liraglutide treated group that may have influenced our primary outcome. Despite, this there are yet no large clinical studies demonstrating any negative action with GLP-1RA on CV risks (2, 24, 25), or hospitalization for heart failure (26). Since GLP-1 receptors only are located near the sinus atrial node (27), the action of increased HR from GLP-1RA may be of direct nature, although a sympathetic influence from GLP-1RA cannot be ruled out.

There are several indirect actions reported on heart function evoked by GLP-1RA (28). These actions may include alterations in the substrate of fatty acids and glucose delivered to the heart, decreased low-grade inflammation in the myocardium, and a reduction in systolic blood pressure (28). T2D commonly associates with the metabolic syndrome in which obesity, elevated levels of triglycerides, low levels of high-density lipoprotein cholesterol, hypertension, and low-grade inflammation (reflected by an increase in CRP levels) are involved. These are all risk factors that may affect myocardial structure and heart function and subsequent development of HF-PEF in subjects with T2D (29). In the current study, T2D patients had the metabolic syndrome, although treated groups were well matched in terms of clinical characteristics and parameters in the metabolic syndrome. Hyperglycemia, reflected by elevated HbA1c, is also an important risk factor for heart failure (30). In the present study, HbA1c was robustly decreased in both groups, with no treatment change between groups; however, treatment change was observed in body weight reduction, in favor for liraglutide, which might have biased our results on heart function (31), in which it should rather have improved diastolic function in the liraglutide treated group (32).

Although mechanisms behind the CV benefits of the LEADER study remains elusive (2), it is suggested related to the modified atherosclerotic process rather than prompt hemodynamic actions, as suggested for the EMPA-REG OUTCOME trial (33). Recent large well-conducted randomized studies have demonstrated neutral effect with GLP-1RA on stabile heart failure (7), or advanced heart failure (34), in patients with and without diabetes. Among patients hospitalized with heart failure, liraglutide treatment did not lead to greater clinical stability (6), a finding concordant with some other small clinical studies showing no beneficial effects from GLP-1RA on heart failure (8, 35). A recent double-blind placebo-controlled crossover study, which used dobutamine stress TDE, to investigate changes in systolic heart function in newly diagnosed T2D patients with coronary artery disease, also failed to show any beneficial action from liraglutide treatment compared with placebo treatment (14). There are, however, some differences between our and recent studies (8, 14, 17, 35) for the investigation of GLP-1RA treatment in T2D patients with heart failure which has to be discussed. First, we investigated T2D patients with HF-PEF with expectation to restore early signs of heart failure; this is in contrast to studies using GLP-1RA treatment into patients with more advanced heart failure (6, 7, 35, 36), or solely systolic heart function (14). Second, we investigated the assessments of LFRI to investigate whether treatment with liraglutide may restore HF-PEF in T2D patients (11). Third, we used sulfonylurea, instead of placebo, to rule out difference in glycemic control as a confounding factor for the primary outcome (32).

In the present study, there are several limitations that have to be discussed. Due to technical hitches and dropouts, 80% (50 out of 62) of patients had full data and was subsequently analyzed per-protocol. Despite this, we believe that current study was powered for our primary endpoint. After multiple imputations (intention-to-treat analysis), the results were not changed supporting the per-protocol analysis. Factors such as transducer position, echocardiography operators (two different sites) may have contributed to some difference. However, due to our pre-specified protocol for the TDE measurements (which also was reflected by the well-matched workload in stress exercise echocardiography between visit 2 and visit 5) and with one blinded off-line investigator make these differences of less concern. Even though the groups were well matched, and well controlled during the study, they might simply have been to healthy for the detection of treatment change, e.g., short diabetes duration, low prevalence of smoking, normal eGFR, and properly controlled hypertension. Also, one major limitation might be too short follow-up study period to detect any treatment change between groups.

In conclusion, 18-week liraglutide treatment did not improve diastolic or systolic LFRI, however increase HR, in T2D patients with subclinical heart failure. There was a robust decrease in HbA1c with no treatment change between groups, and a significant treatment change in body weight reduction in favor for liraglutide treatment. Evidence of GLP-1RA treatment on heart failure are mixed. Since LEADER trial show beneficial effect on CV outcome further clinical studies should be launched to investigate more in detail what mechanisms are behind these results.

## Ethics Statement

All patients provided written informed consent to participate in the study, which was conducted in accordance with the Declaration of Helsinki and Good Clinical Practice. The research project was approved by the Ethics Committee of Stockholm Sweden, and the Swedish Medical Products Agent. Moreover, the study was registered in http://www.clinicaltrials.gov. Unique identifier: NCT01425580.

## Author Contributions

All authors contributed to the study conception and design. TN and IS analyzed data and wrote the first draft of the paper. All authors commented and took part of the revision of the paper. TN is the guarantor of the manuscript.

## Conflict of Interest Statement

TN has received unrestricted grants from AstraZenca and consultancy fees from Boehringer Ingelheim, Eli Lilly, Novo Nordisk, Merck, and Sanofi-Aventis. JJ has received consultancy fees from Boehringer Ingelheim, Eli Lilly, NovoNordisk, Medtronic, Menarini Diagnostics, MSD, and Roche. No other disclosures were reported. The reviewer JB and handling editor declared their shared affiliation.
