# Corrigendum: Effects on Subclinical Heart Failure in Type 2 Diabetic Subjects on Liraglutide Treatment vs. Glimepiride Both in Combination with Metformin: A Randomized Open Parallel-Group Study

**DOI:** 10.3389/fendo.2018.00050

**Published:** 2018-02-22

**Authors:** Thomas Nyström, Irene Santos-Pardo, Fredric Hedberg, Johan Wardell, Nils Witt, Yang Cao, Leif Bojö, Bo Nilsson, Johan Jendle

**Affiliations:** ^1^Department of Clinical Science and Education, Karolinska Institutet, Södersjukhuset, Stockholm, Sweden; ^2^Institution of Medical Sciences, Örebro University, Örebro, Sweden; ^3^Unit of Biostatistics, Institute of Environmental Medicine, Karolinska Institutet, Stockholm, Sweden; ^4^Central Hospital, Karlstad, Sweden; ^5^Clinical Epidemiology and Biostatistics, School of Medical Sciences, Örebro University, Örebro, Sweden

**Keywords:** longitudinal functional reserve index, liraglutide, subclinical heart failure, tissue Doppler echocardiography, type 2 diabetes

One of the authors name, Irene Santos-Pardo, was misspelled in the published manuscript (as Irene Padro Santos). The authors apologize for the mistake. This error does not change the scientific conclusions of the article in any way.

The original article was updated.

## Conflict of Interest Statement

The authors declare that the research was conducted in the absence of any commercial or financial relationships that could be construed as a potential conflict of interest.

